# Some Account of the Efficacy of Tight Bandages and Oil of Turpentine, Applied to the Extremities in Certain Stages of Autumnal Fever

**Published:** 1829

**Authors:** William Maclay Awl

**Affiliations:** Somerset, Ohio


*November*, 1829.

---

Art. III.—*Some account of the efficacy of tight bandages and oil of turpentine, applied to the extremities in certain stages of Autumnal Fever.* By William Maclay Awl, M. D. of Somerset, Ohio.

Every autumn presents cases or stages of fever, which are termed congestive. The heart fails to throw the blood to the surface and the fluid stagnates in the great trunks; the bloodless extremities shrink up, and the hands resemble those of a washerwoman while engaged in her calling; the skin loses its sensibility, and heat, and is covered with cold perspiration; the faculties of sensation and volition are greatly impaired, the countenance becomes Hippocratic, and death seems—in fact generally is, impending. In this sad condition, internal stimulants, external irritation, frictions, and culinary heat are resorted to, but in most cases without effect.

In the summer of 1829, I aws called to see a labourer on the Canal, who had been hauled 20 miles in a wagon under a burning sun, vomiting the whole way. His limbs were cold, and covered with a profuse sweat. Death, indeed, seemed to be at hand. Sitting by his bed side, and reflecting on the most proper course to be pursued, I rubbed one of his almost lifeless arms, till the perspiration was dried up, and the skin acquired a little colour when I covered it up. But in 10 minutes it was colder than ever, and literally deluged in perspiration, as if the integuments had entirely lost their containing power. An observation of this fact, suggested to my mind the propriety, and possible advantage of bandaging his extremities, and I immediately proceeded to verify my speculation by reducing it to practice. Procuring a sufficient number of flannel rollers, I bandaged, tightly and neatly, the whole of his limbs from their extremities to the trunk of the body, and moistening them with spirit of turpentine, left him to his fate. In six hours he was a new creature. His countenance was revived, his pulse had risen, and the heat and colour of his limbs were restored. Regular paroxysms of autumnal fever supervened, which being met with the usual remedies, he recovered.

I have since the above case, used the bandages repeatedly, and always with success, sometimes as in the same case having to trust to it alone, the situation of the stomach preventing other means, and at other times using it both as an adjuvant, and a preventive remedy. And further, I have extended its application to various other adynamic prostrations of the system; particularly that attendant on cholera morbus, in which disease I constantly make it an auxiliary to the common means, when the case is severe. I have no doubt that it would contribute signally to the preservation of life in many cases of extreme capillary exhaustion, and might be found singularly useful in yellow fever.

The following is an extract from a letter received from Dr. Thomas H. Gibson, of Circleville, in this state, one of my correspondents, upon the subject under consideration. "I thank you a thousand times for your idea of bandages in certain states of extreme capillary exhaustion. I saved the father of a large and helpless family, by their use after every thing else had failed. It was a case of cholera, aggravated by his having taken a large portion of gamboge, colomel, and tartar emetic pills. After having spent two or three hours, fruitlessly, in endeavoring to remove cold extremities, præcordial anxiety, difficulty of breathing, and other symptoms which portend dissolution, by ordinary and extraordinary stimulants; I ripped up a wollen frock and incased his extremities, and the result was delightfully successful. I wonder that I should not have thought of it long ago. It is the very thing I have wanted for years. It is precisely applicable, to those cases in which medicines have no longer any effect. Applied at the right time, it would save 7 in 10 of those who die of our western autumnal diseases."

*November*, 1829.

